# Overcoming Multidrug Resistance in *Salmonella* spp. Isolates Obtained From the Swine Food Chain by Using Essential Oils: An *in vitro* Study

**DOI:** 10.3389/fmicb.2021.808286

**Published:** 2022-02-09

**Authors:** Carlotta Lauteri, Francesca Maggio, Annalisa Serio, Anna Rita Festino, Antonello Paparella, Alberto Vergara

**Affiliations:** ^1^Section of Food Inspection, Faculty of Veterinary Medicine, School of Specialization in Inspection of Foods of Animal Origin, “G. Tiecco” University of Teramo, Teramo, Italy; ^2^Section of Food Microbiology, Faculty of Bioscience and Technology for Food, Agriculture and Environment, University of Teramo, Teramo, Italy

**Keywords:** antimicrobial resistance, *Salmonella* spp., essential oil, *Thymus vulgaris*, *Eugenia caryophyllata*, *Corydothymus capitatus*, tetracycline, swine production chain

## Abstract

Antimicrobial resistance (AMR) is a global concern, and new approaches are needed to circumvent animal and food-borne resistant pathogens. Among the new strategies, the combination of antibiotics with natural compounds such as essential oils (EOs) could be an alternative to challenge bacterial resistance. The present study evaluates the phenotypic and genotypic antibiotic resistance of 36 *Salmonella enterica* (16 *S.* Typhimurium, 3 monophasic variant *S.* Typhimurium, 8 *S.* Enteritidis, 6 *S.* Rissen, 1 *S.* Typhi, and 2 *S.* Derby) strains, isolated from the swine production chain. The isolates displayed phenotypic resistance to gentamicin, amikacin, tobramycin, and tetracycline, while the resistance genes most commonly detected were *parC*, *catA*, *nfsB*, *nfsA*, *blaTEM*, *tetA*, and *tetB*. Then 31/36 *Salmonella* isolates were chosen to evaluate resistance to tetracycline and *Thymus vulgaris*, *Eugenia caryophyllata*, and *Corydothymus capitatus* EOs by determining minimum inhibitory concentrations (MICs). Finally, the synergistic effect between tetracycline and each EOs was evaluated by the checkerboard method, calculating the fractional inhibitory concentration (FIC) index. Among the EOs, *C. capitatus* displayed the best bioactivity in terms of MICs, with the lowest values (0.31 and 0.625 μl/ml). On the contrary, the strains showed the ability to grow in the presence of the maximum concentration of tetracycline employed (256 μg/ml). While not displaying a real synergism according to the FIC index, the combination of tetracycline compounds and the three EOs resulted in a significant reduction in the MIC values to tetracycline (4 μg/ml), suggesting a restoration of the susceptibility to the antibiotic in *Salmonella* spp.

## Introduction

*Salmonella* spp. is widespread in the environment, but the main reservoir is the intestinal tract of livestock animals and particularly pig, poultry, and cattle. The pathogen can be transmitted to humans through the food chain (Prasertsee et al., 2016). In Europe and in the United States, contaminated pork and pork products are important sources for salmonellosis in humans (Broadway et al., 2021; EFSA, 2021). *Salmonella* infections in humans can be divided in two main forms, including invasive typhoidal salmonellosis and non-typhoidal salmonellosis. The former, caused by *S. enterica* (serotype Typhi and Paratyphi A, B), causes enteric fever, gastroenteritis, and bacteremia. The latter can be caused by several *Salmonella* serovars. Among these, non-typhoid serotypes, such as *S. enterica* Typhimurium, has a broad vertebrate host range and causes various symptoms that usually include diarrheal disease (Andrews and Ryan, 2015).

On the other hand, salmonellosis in swine is caused by ubiquitous *Salmonella* serovars that can occur as a symptomatic disease in a wide range of hosts and, more frequently, as a self-limiting gastroenteritis. Typical symptoms in pigs are enteric, but infected animals are frequently asymptomatic (Bonardi et al., 2016). The presence of infected pigs that acquire a healthy carrier status may pose a threat to public health (Bonardi et al., 2016) and can lead to cross-contamination of carcasses. For this reason, during pig production and particularly in lactation and post-weaning, an extensive administration of oral antibiotics, such as penicillin and tetracycline, occurs (Lekagul et al., 2019). Nevertheless, the use of antimicrobials either to treat or to prevent infections, as well as growth promoters in farm animals, is a major contributing factor for the development of antimicrobial resistance (AMR), potentially leading to the widespread transmission of antimicrobial-resistant bacteria through the food chain (Andrews and Ryan, 2015). Therefore, *Salmonella* spp. displays the capability of spreading antibiotic resistance by *transfer-*associated genes, thus, causing the increase in incidence and severity of the disease. In fact, treatment of salmonellosis in humans and animals has become more difficult due to the emergence of multidrug-resistant *Salmonella* spp. strains (Lekagul et al., 2019).

Antimicrobial resistance is defined as a biological phenomenon of adaptation of some microorganisms that acquire the ability to survive or grow in the presence of an antimicrobial agent (Palma et al., 2020). The ability to resist is due to genetic mutations or acquisition *via* lateral gene transfer of resistance genes. Despite subsequent restrictions and bans on the use of different antimicrobials in agriculture, human and veterinary medicine, the resistance acquired by the microorganism is retained and is potentially transmissible (World Health Organization [WHO], 2020). Nowadays, alternative treatments to counteract AMR have been evaluated, for example, the use of natural compounds such as essential oils (EOs) (Trifan et al., 2020; Maggio et al., 2021). EOs are oily systems containing a mixture of different bioactive molecules derived by aromatic plants (Rossi et al., 2020). The phytocomplex contained in EOs interacts with multiple bacterial cellular targets, instead of adopting a particular single mode of action, thus, preventing pathogens from acquiring resistance (Yang et al., 2018). EO antimicrobial efficacy is associated with the main compounds; however, the EOs are a consortium of different compounds, each with its own effectiveness. For example, bioactive monoterpenes, such as thymol and carvacrol, which are found mainly in *Origanum* and *Thymus* spp. EOs, possess the ability to destabilize the outer membrane of Gram-negative bacteria, causing an increase in membrane permeability that is a mechanism of antimicrobial action (Lambert et al., 2001). Moreover, phenylpropanoids, such as eugenol, frequently found in clove EO (Moemenbellah-Fard et al., 2020), can modify the fatty acid profile of the cell membrane (Marchese et al., 2017). The destabilization of the cell membrane increases the susceptibility of the bacteria toward other antimicrobial compounds. Therefore, the combination of an antibiotic treatment with EOs could allow the natural compounds to permeate the cell membrane under the action of antibiotics, reducing the concentration of the bioactive compounds employed (Yang et al., 2018) and restoring bacterial susceptibility to treatments.

Considering the above reasons, this study first aimed at evaluating the resistance of 36 *Salmonella* spp. strains from the swine production chain to different antibiotics, generally employed in livestock. Afterward, the potential effect of EOs in restoring the susceptibility of the strains to the antibiotics was investigated. In particular, a selection of tetracycline-resistant isolates was subjected to a combination of different antibiotics and *Corydothymus capitatus*, *Eugenia caryophyllata*, and *Thymus vulgaris* EOs.

## Materials and Methods

### Bacterial Strains

A total of 36 *Salmonella* spp. (16 *S.* Typhimurium, 3 monophasic variant *S.* Typhimurium, 8 *S.* Enteritidis, 6 *S.* Rissen, 1 *S.* Typhi, and 2 *S.* Derby) strains, belonging to the biobank of the Department of Food Inspection of the University of Teramo (Italy), previously isolated from the swine production chain and identified, were included in this study (Di Ciccio et al., 2016; Lauteri et al., 2021).

### Antimicrobial Susceptibility Testing

The card VITEK 2 AST GN65 was used according to the instructions of the manufacturer (bioMérieux, 2013a) to evaluate antibiograms and determine minimum inhibitory concentrations (MICs). Fifteen antimicrobial agents were tested, in detail: ampicillin, amoxicillin and clavulanic acid, imipenem, cefpodoxime, ceftiofur, tobramycin, piperacillin, gentamicin, amikacin, enrofloxacin, marbofloxacin, chloramphenicol, tetracycline, nitrofurantoin, and trimethoprim-sulfamethoxazole.

The turbidity of the bacterial suspensions was adjusted with a densitometer (DENSICHEK, bioMerieux, Marcy-l’Etoile, France) to match a 0.5–0.63 McFarland standard; then 145 μl of suspension was added to 3 ml of VITEK 0.45% saline solution (bioMérieux, 2013b). The time range between suspension preparation and card filling was less than 30 min to avoid changes in turbidity.

Afterward, the VITEK 2 AST GN65 antimicrobial susceptibility cards and bacterial suspension in tubes, both contained in a cassette, were manually loaded into the VITEK 2 system. Each test card was automatically filled with a bacterial suspension, sealed, incubated, and read by kinetic fluorescence measurement. The reporting time for the direct testing of susceptibility against the 15 antibiotics for culture isolates by the VITEK 2 system ranged from 8.5 to 10.5 h.

### Detection of Antibiotic Resistance Genes

The presence of AMR genes was investigated using conventional polymerase chain reaction (PCR) (Kikuvi et al., 2010). The uniplex PCR amplification conditions consisted of initial denaturation at 94°C for 5 min, with 30 cycles of denaturation at 94°C for 30 s, annealing at different temperatures according to the different primers for 30 s, extension at 72°C for 1 min, and a final cycle of amplification at 72°C for 10 min. The specific primers used in the PCR amplifications, the annealing temperatures, and the amplicon sizes are reported in Table 1. PCR products were resolved by agarose gel electrophoresis.

**TABLE 1:** Target antibiotic, genes, PCR primers, forward and reverse sequence, annealing temperature of the primers, amplicon size, and reference used to evaluate the presence of antibiotic-resistant genes.

Antibiotic	Gene	Sequence (5′–3′)	Annealing temperature (°C)	Amplicon size (bp)	References
Tetracycline	*tetA*	F-GTAATTCTGAGCACTGT R-CCTGGACAACATTGCTT	45	954	Kikuvi et al., 2010
Tetracycline	*tetB*	F-ACGTTACTCGATGCCAT R-AGCACTTGTCTCCTGTT	48	1,170	Kikuvi et al., 2010
Tetracycline	*tetC*	F-AACAATGCGCTCATCGT R-GGAGGCAGACAAGGTAT	50	1,138	Kikuvi et al., 2010
Chloramphenicol	*catA1*	F-GGCATTTCAGTCAGTTG R-CATTAAGCATTCTGCCG	50	551	Kikuvi et al., 2010
Aminoglycosides	*aadA2*	F-CGGTGACCATCGAAATTTCG R-CTATAGCGCGGAGCGTCTCGC	54	250	Prasertsee et al., 2016
Aminoglycosides	*aac(3)IV*	F-TGCTGGTCCACAGCTCCTTC R- CGGATGCAGGAAGATCAA	63	653	Kozak et al., 2009
Aminoglycosides	*aadB*	F-GAGGAGTTGGACTATGGATT R-CTTCATCGGCATAGTAAAAG	55	208	Kozak et al., 2009
Ampicillin	*bla*TEM	F-CCGTGTCGCCCTTATTCCC R-GCCTGACTCCCCGTCGTGT	51	780	Kikuvi et al., 2010
Ampicillin	*bla*PSE	F-CGCTTCCCGTTAACAAGTAC R-CTGGTTCATTTCAGATAGCG	58	465	Kikuvi et al., 2010
Nitrofurantoin	*nfsA*	F-CTGGCGCTTGCTCTGCTATC R-GCCCGAGTATCATACACTGG	60	964	Garcia et al., 2016
Nitrofurantoin	*nfsB*	F-ACTACCGTCTCGCTACTCAAC R-CGCGCCATTGATCATTGAGG	58	921	Garcia et al., 2016
Quinolone	*parC*	F-CTATGCGATGTCAGAGCTGG R-TAACAGCAGCTCGGCGTATT	62	270	El-Tayeb et al., 2017

### Inocula and Growth Media

For the subsequent analyses, 31 *Salmonella* strains (15 *S.* Typhimurium, 3 monophasic variant *S.* Typhimurium, 6 *S.* Enteritidis, 6 *S.* Rissen, and 1 *S.* Typhi) were selected on account of their resistance to tetracycline, as described before. The inocula were prepared in Mueller–Hinton broth (MH, Oxoid Thermo Fisher Scientific, Rodano, Milan, Italy) and incubated at 37°C for 18 h until early stationary phase. Cells were then harvested by centrifugation and washed three times with phosphate buffer saline (PBS) 50 mM, pH 7.4. The inocula were standardized to OD_620__nm_ 0.1–0.2 (5 × 10^7^ cells/ml) and then diluted to 5 × 10^6^ cells/ml.

### Antimicrobial Solutions

Commercial and food-grade *T. vulgaris* and *E. caryophyllata* EOs were kindly provided by Flora S.r.l. (Pisa, Italy), while *C. capitatus* EO was supplied by Exentiae S.r.l. Soc. Agricola (Catania, Italy). According to the results from the analyses carried out by the producers, the EO chemotypes were thymol (46.65%), eugenol (76.2%), and carvacrol (70%), for *T. vulgaris*, *E. caryophyllata*, and *C. capitatus*, respectively. EO emulsions were diluted to 80 μl/ml in PBS and 1% Tween 80 (Sigma-Aldrich, Milan, Italy). Lyophilized tetracycline (≥98%) was provided by Sigma-Aldrich (Milan, Italy).

### Minimum Inhibitory Concentration/Minimum Bactericidal Concentration Assays

The MICs were determined for the EO emulsions and the antibiotic solutions following the CLSI guidelines/CLSI protocol (CLSI, 2016) in a 96-well microtiter plate (Corning Incorporated, Kennebunk, ME, United States). The antibacterial activity was examined after incubation at 37°C for 72 h in static conditions. MICs were determined after 24, 48, and 72 h, evidenced by the absence of red discoloration by 2,3,5-triphenyltetrazolium chloride (Sigma-Aldrich, Milan, Italy), added in the growth media in a ratio of 0.1%. Subsequently, the minimum bactericidal concentration (MBC) was determined after 24, 48, and 72 h at 37°C by plating out onto MH agar plates.

### Checkerboard Test

The synergy between tetracycline and each EO was tested by the checkerboard method, a two-dimensional matrix of serial concentrations of the compounds under examination (Magi et al., 2015). By the checkerboard test, it was possible to calculate a fractional inhibitory concentration (FIC) index, according to the formulas (Magi et al., 2015):


$$F\,I\,C_{A}=M\,I\,C_{A+B}/M\,I\,C_{A},$$

$$F\,I\,C_{B}=M\,I\,C_{B+A}/M\,I\,C_{B},$$

$$F\,I\,C\,I\,n\,d\,e\,x=F\,I\,C_{A}+F\,I\,C_{B}.$$

Moreover, the FIC index values were interpreted in agreement with Fratini et al. (2017): synergistic effect (FIC index ≤ 1.0), commutative effect (FIC index = 1), no interaction (FIC index > 1.0–2.0), and antagonistic effect (FIC index > 2.0).

### Statistical Analysis

The data of MIC and MBC assays were expressed as the means of three different repetitions. The datasets of the MICs of tetracycline (μg/ml) from the *in vitro* analysis in combination with the three EOs (μl/ml) and alone were correlated through the principal component analysis (PCA), using the XLSTAT 2014 software (Redmond, WA, United States).

## Results

### Antimicrobial Susceptibility of *Salmonella* Isolates

All *Salmonella* isolates selected for this study displayed resistance to gentamicin, amikacin, and tobramycin.

A total of 86.1% (31/36) of the *Salmonella* isolates were resistant to tetracycline, while 55.5% (20/36) were resistant to ampicillin and piperacillin. Twenty-five percent (9/36) of the strains showed resistance to trimethoprim, 5.5% (2/36) to chloramphenicol, and only 2.8% (1/36) to amoxicillin–clavulanic acid and nitrofurantoin.

Moreover, 16 *Salmonella* isolates (44.4%) showed intermediate resistance to nitrofurantoin and 36.1% (13/36) to chloramphenicol. A total of 8.3% (3/36) of the isolates exhibited intermediate resistance against amoxicillin–clavulanic acid, while 5.5% (2/36) had intermediate resistance to enrofloxacin and 2.8% (1/36) to ceftiofur.

Several strains showed multiple AMR, namely, 27.8% (10/36) of the strains had resistance to three antimicrobial classes, 25% (9/36) were resistant to four antimicrobial classes, and 2.8% (1/36) displayed resistance to even six antimicrobial classes.

The results of the antimicrobial susceptibility testing are reported in Table 2.

**TABLE 2:** Overview of the antibiotic resistance shown by the 36 investigated isolates of *Salmonella* spp.

Strain	Serovar	Resistance phenotype	Resistance genotype
114	*S.* Typhimurium	AMI, AMP, GEN, PIP, TOB	*parC*
115	*S.* Typhimurium	AMI, AMP, GEN, PIP, TET, TOB, TRI	*catA1*, *parC*, *tetA*, *tetB*
117	*S.* Typhimurium	AMI, GEN, TET, TOB	*catA1*, *nfs B*, *parC*, *tetA*, *tetB*
118	*S.* Typhimurium	AMI, GEN, TET, TOB	*catA1*, *nfsA*, *parC*, *tetB*
669	*S.* Typhimurium	AMI, AMP, GEN, PIP, TET, TOB	*catA1*, *nfsA*, *nfs B*, *parC*, *tetB*
670	*S.* Typhimurium	AMI, GEN, TET, TOB	*blaTEM*, *catA1*, *nfsA*, *nfs B*, *parC*, *tetA*
685	*S.* Typhimurium	AMI, GEN, TET, TOB	*catA1*, *nfsA*, *nfs B*, *parC*, *tetA*
686	*S.* Typhimurium	AMI, GEN, TET, TOB	*catA1*, *nfsA*, *nfs B*, *parC*, *tetA*
687	*S.* Typhimurium	AMI, AMP, GEN, PIP, TET, TOB	*blaTEM*, *catA1*, *nfsA*, *nfsB*, *parC*, *tetB*
689	*S.* Typhimurium	AMI, AMP, GEN, PIP, TET, TOB	*blaTEM*, *catA1*, *nfsA*, *nfsB*, *parC*, *tetB*
690	*S.* Typhimurium	AMI, GEN, TET, TOB	*catA1*, *nfsA*, *nfsB*, *parC*, *tet C*
691	*S.* Typhimurium	AMI, AMP, GEN, PIP, TET, TOB	*blaTEM*, *catA1*, *nfsA*, *nfsB*, *parC*, *tetB*
693	*S.* Typhimurium	AMI, AMP, GEN, PIP, TET, TOB	*blaTEM*, *catA1*, *nfsA*, *nfsB*, *parC*, *tetB*
694	*S.* Typhimurium	AMI, AMP, GEN, PIP, TET, TOB	*blaTEM*, *catA1*, *nfsA*, *nfsB*, *parC*, *tetB*
695	*S.* Typhimurium	AMI, AMP, GEN, PIP, TET, TOB	*blaTEM*, *catA1*, *nfsA*, *nfsB*, *parC*, *tetB*
785	*S.* Typhimurium	AMI, AMP, AMX, CEF, CLO, GEN, NIT, PIP, TET, TOB	*catA1*, *parC*, *tetB*
787	Monophasic variant *S.* Typhimurium	AMI, AMP, GEN, PIP, TET, TOB	*aadB*, *blaTEM*, *catA1*, *nfsA*, *nfsB*, *parC*, *tetB*
791	Monophasic variant *S.* Typhimurium	AMI, AMP, GEN, PIP, TET, TOB	*aadB*, *blaTEM*, *catA1*, *nfsA*, *nfsB*, *parC*, *tetB*
792	Monophasic variant *S.* Typhimurium	AMI, AMP, GEN, PIP, TET, TOB	*blaTEM*, *nfsA*, *nfsB*, *parC*, *tetB*
205	*S.* Enteritidis	AMI, AMP, GEN, PIP, TET, TOB, TRI	*blaTEM*, *catA1*, *nfsA*, *nfsB*, *parC*, *tetA*
206	*S.* Enteritidis	AMI, AMP, GEN, PIP, TET, TOB, TRI	*blaTEM*, *catA1*, *nfsA*, *nfsB*, *parC*, *tetA*
207	*S.* Enteritidis	AMI, AMP, GEN, PIP, TET, TOB, TRI	*blaTEM*, *catA1*, *nfsA*, *nfsB*, *parC*, *tetA*
208	*S.* Enteritidis	AMI, AMP, GEN, PIP, TET, TOB, TRI	*blaTEM*, *catA1*, *nfsB*, *parC*, *tetA*
216	*S.* Enteritidis	AMI, AMP, GEN, PIP, TET, TOB, TRI	*blaTEM*, *catA1*, *nfsA*, *nfsB*, *parC*, *tetA*
217	*S.* Enteritidis	AMI, AMP, GEN, PIP, TET, TOB, TRI	*blaTEM*, *catA1*, *nfsB*, *parC*, *tetA*
671	*S.* Enteritidis	AMI, GEN, TOB	*catA1*, *nfsA*, *nfsB*, *parC*
688	*S.* Enteritidis	AMI, GEN, TOB	*catA1*, *nfsA*, *nfsB*, *parC*
116	*S.* Typhi	AMI, AMP, GEN, PIP, TET, TOB, TRI	*catA1*, *blaTEM*, *parC*, *tetA*, *tetB*
122	*S.* Rissen	AMI, GEN, TET, TOB	*catA1*, *nfsA*, *nfsB*, *parC*
786	*S.* Rissen	AMI, GEN, TET, TOB	*catA1*, *nfsA*, *nfsB*, *parC*, *tetA*
788	*S.* Rissen	AMI, GEN, TET, TOB	*aac(3)IV*, *catA1*, *nfsB*, *parC*, *tetA*
790	*S.* Rissen	AMI, GEN, TET, TOB	*catA1*, *nfsA*, *nfsB*, *parC*, *tetA*
793	*S.* Rissen	AMI, CLO, GEN, TET, TOB, TRI	*catA1*, *nfsA*, *nfsB*, *parC*, *tetA*
795	*S.* Rissen	AMI, GEN, TET, TOB	*catA1*, *nfsA*, *nfsB*, *parC*, *tetA*
789	*S.* Derby	AMI, GEN, TOB	*catA1*, *nfsA*, *nfsB*, *parC*
794	*S.* Derby	AMI, GEN, TOB	*catA1*, *nfsA*, *nfsB*, *parC*

*AMI, amikacin; AMP, ampicillin; AMX, amoxicillin–clavulanic acid; CEF, ceftiofur; CLO, chloramphenicol; GEN, gentamicin; NIT, nitrofurantoin; PIP, piperacillin; TETRA, tetracycline; TOB, tobramycin; TRI, trimethoprim-sulfamethoxazole.*

### Detection of Antibiotic Resistance Genes

All *Salmonella* isolates recovered from the swine production chain were investigated for the presence of AMR genes by PCR. The most frequently detected resistance genes were *parC* (36/36, 100%), *catA1* (34/36, 94.4%), *nfsB* (31/36, 86.1%), *nfsA* (28/36, 77.7%), *blaTEM* (17/36, 47.2%), *tetA* (17/36, 47.2%), and *tetB* (15/36, 41.6%). The genes *tetC*, *aac(3)IV*, and *aadB* were detected only in one isolate, corresponding to 2.8% of the total, while *aadA2* and *blaPSE* were not detected. The association of phenotypic resistance and the presence of AMR genes was variable among the different *Salmonella* serovars. For example, while phenotypic resistance to one or more antimicrobials was observed for both *S.* Derby (*n* = 2) and *S.* Rissen (*n* = 5), the corresponding resistance genes were not detected by PCR. On the contrary, *S.* Rissen isolate 122, which tested positive for the presence of *catA1*, *nfsA*, *nfsB*, and *parC*, did not show any AMR (Table 2).

### Minimum Inhibitory Concentration and Minimum Bactericidal Concentration Determination of Tetracycline and Essential Oils

Minimum inhibitory concentrations and MBCs for tetracycline and EOs were determined after 48 h of incubation at 37°C by broth microdilution assay (Table 3). The MIC of tetracycline was 256 μg/ml for each *Salmonella* strain. According to the Clinical and Laboratory Standards Institute guidelines (CLSI guidelines, supplement M100S), the strains were classified as resistant to tetracyclines [as per the MIC breakpoints for tetracycline, strain result susceptible (≤4 μg/ml), intermediate (8 μg/ml), and resistant (≥16 μg/ml)]. Regarding the MICs of the EOs, *C. capitatus* displayed the best bioactivity, with a range of values between <0.31 and 10 μl/ml. In particular, the lowest MIC values were those observed more frequently in the strains analyzed and, in detail, 0.31 μl/ml for 5 isolates (16.1%) and 0.625 μl/ml for 23 isolates (74.2%) out of 31. Nevertheless, *T. vulgaris* EO exhibited the lowest MIC values ranging from <0.31 to 5 μl/ml, although the MIC values observed more frequently were 0.625 and 1.25 μl/ml, with 7 (22.6%) and 18 (58%) isolates out of 31, respectively. *E. caryophyllata* EO showed the lowest effectiveness, with MIC values between 0.31 and 20 μl/ml. Moreover, the highest MIC values were common in the set of strains, with 10 and 20 μl/ml for 6 (19.3%) and 16 isolates out of 31 (51.6%), respectively.

**TABLE 3:** Minimum inhibitory concentration (MIC), MBC values, and FIC index of *Coridothymus capitatus*, *Eugenia caryophyllata* and *Thymus vulgaris* EOs alone and in combination with tetracycline against *Salmonella* strains, determined by broth dilution technique and checkerboard method after 48 h of incubation at 37°C.

	MIC							MBC							FIC_*A*_			FIC_*B*_	FICI			Activity		
Strain	C	G	T	Tc	C-Tc	G-Tc	T-Tc	C	G	T	Tc	C-Tc	G-Tc	T-Tc	C	G	T	Tc	C	G	T	C	G	T
115	0.625	1.25	1.25	>256	0.625–4	0.625–4	1.25–4	1.25	20	1.25	>256	1.25–4	20–4	1.25–4	1	0.5	1	0.02	1.0	0.5	1.0	C	S	C
116	5	10	2.5	>256	5–4	10–4	2.5–4	1.25	1.25	1.25	>256	1.25–4	1.25–4	1.25–4	1	1	1	0.02	1.0	1.0	1.0	C	C	C
117	0.625	20	5	>256	0.625–4	10–4	2.5–4	10	0.31	1.25	>256	10–4	2.5–4	1.25–4	1	0.5	0.5	0.02	1.0	0.5	0.5	C	S	S
118	1.25	20	5	>256	0.625–4	10–4	5–4	5	10	2.5	>256	10–4	10–4	2.5–4	0.5	0.5	1	0.02	0.5	0.5	1.0	S	S	C
122	2.5	20	2.5	>256	5–4	20–4	2.5–4	0.625	20	2.5	>256	0.625–4	10–4	2.5–4	2	1	1	0.02	2.0	1.0	1.0	I	C	C
205	0.625	2.5	1.25	>256	0.625–4	2.5–4	1.25–4	1.25	20	2.5	>256	0.625–4	20–4	5–4	1	1	1	0.02	1.0	1.0	1.0	C	C	C
206	0.625	2.5	0.625	>256	0.625–4	2.5–4	0.625–4	2.5	20	2.5	>256	5–4	20–4	2.5–4	1	1	1	0.02	1.0	1.0	1.0	C	C	C
207	1.25	10	0.625	>256	1.25–4	10–4	0.625–4	0.31	1.25	1.25	>256	0.31–4	1.25–4	1.25–4	1	1	1	0.02	1.0	1.0	1.0	C	C	C
208	0.625	2.5	1.25	>256	0.625–4	2.5–4	0.625–4	0.31	2.5	0.625	>256	0.31–4	2.5–4	0.625–4	1	1	0.5	0.02	1.0	1.0	0.5	C	C	S
216	1.25	1.25	0.31	>256	0.625–4	1.25–4	0.31–4	0.625	5	1.25	>256	1.25–4	5–4	1.25–4	0.5	1	1	0.02	0.5	1.0	1.0	S	C	C
217	0.625	1.25	0.625	>256	0.625–4	1.25–4	1.25–4	0.625	2.5	1.25	>256	0.625–4	2.5–4	1.25–4	1	1	2	0.02	1.0	1.0	2.0	C	C	I
669	0.625	2.5	1.25	>256	0.625–4	2.5–4	1.25–4	0.625	5	1.25	>256	0.625–4	2.5–4	1.25–4	1	1	1	0.02	1.0	1.0	1.0	C	C	C
670	0.625	2.5	1.25	>256	0.625–4	2.5–4	1.25–4	0.625	2.5	0.625	>256	0.625–4	2.5–4	0.625–4	1	1	1	0.02	1.0	1.0	1.0	C	C	C
685	0.625	2.5	1.25	>256	0.625–4	2.5–4	1.25–4	0.625	1.25	0.625	>256	0.625–4	1.25–4	0.625–4	1	1	1	0.02	1.0	1.0	1.0	C	C	C
686	1.25	5	0.625	>256	1.25–4	5–4	1.25–4	0.625	0.625	1.25	>256	1.25–4	1.25–4	1.25–4	1	1	2	0.02	1.0	1.0	2.0	C	C	I
687	0.625	0.625	1.25	>256	0.625–4	1.25–4	1.25–4	0.625	1.25	1.25	>256	0.625–4	1.25–4	1.25–4	1	2	1	0.02	1.0	2.0	1.0	C	I	C
689	0.625	1.25	1.25	>256	0.625–4	1.25–4	1.25–4	0.625	2.5	2.5	>256	0.625–4	2.5–4	2.5–4	1	1	1	0.02	1.0	1.0	1.0	C	C	C
690	0.31	2.5	0.625	>256	0.625–4	2.5–4	0.625–4	0.625	2.5	1.25	>256	1.25–4	2.5–4	1.25–4	2.02	1	1	0.02	2.0	1.0	1.0	I	C	C
691	0.625	1.25	0.625	>256	0.625–4	1.25–4	0.625–4	1.25	1.25	0.625	>256	1.25–4	5–4	1.25–4	1	1	1	0.02	1.0	1.0	1.0	C	C	C
693	0.31	1.25	1.25	>256	0.31–4	1.25–4	1.25–4	0.625	1.25	0.625	>256	0.625–4	1.25–4	1.25–4	1	1	1	0.02	1.0	1.0	1.0	C	C	C
694	0.31	2.5	0.625	>256	0.31–4	2.5–4	0.625–4	0.625	5	1.25	>256	0.625–4	2.5–4	1.25–4	1	1	1	0.02	1.0	1.0	1.0	C	C	C
695	0.625	5	1.25	>256	1.25–4	5–4	1.25–4	2.5	10	0.625	>256	1.25–4	10–4	0.625–4	2	1	1	0.02	2.0	1.0	1.0	I	C	C
785	10	0.31	1.25	>256	10–4	2.5–4	1.25–4	1.25	5	1.25	>256	1.25–4	5–4	0.625–4	1	4.03	1	0.02	1.0	4.1	1.0	C	A	C
786	1.25	1.25	1.25	>256	1.25–4	1.25–4	1.25–4	1.25	1.25	0.625	>256	0.625–4	1.25–4	0.625–4	1	1	1	0.02	1.0	1.0	1.0	C	C	C
787	<0.31	<0.31	<0.31	>256	0.625–4	1.25–4	0.625–4	0.625	2.5	0.625	>256	0.625–4	5–4	0.625–4	4.03	8.06	4.03	0.02	4.1	8.1	4.1	A	A	A
788	1.25	1.25	1.25	>256	1.25–4	1.25–4	1.25–4	<0.31	<0.31	0.625	>256	1.25–4	1.25–4	1.25–4	1	1	1	0.02	1.0	1.0	1.0	C	C	C
790	0.31	2.5	1.25	>256	0.31–4	2.5–4	1.25–4	1.25	1.25	1.25	>256	1.25–4	1.25–4	1.25–4	1	1	1	0.02	1.0	1.0	1.0	C	C	C
791	1.25	20	1.25	>256	1.25–4	20–4	1.25–4	0.625	2.5	1.25	>256	1.25–4	5–4	1.25–4	1	1	1	0.02	1.0	1.0	1.0	C	C	C
792	0.625	2.5	1.25	>256	1.25–4	2.5–4	1.25–4	0.625	20	1.25	>256	1.25–4	20–4	1.25–4	2	1	1	0.02	2.0	1.0	1.0	I	C	C
793	0.625	20	1.25	>256	1.25–4	20–4	1.25–4	2.5	20	1.25	>256	1.25–4	20–4	1.25–4	2	1	1	0.02	2.0	1.0	1.0	I	C	C
795	1.25	20	1.25	>256	1.25–4	20–4	1.25–4	1.25	1.25	1.25	>256	0.625–4	0.625–4	1.25–4	1	1	1	0.02	1.0	1.0	1.0	C	C	C

*The results of MIC and MBC are expressed as μl/ml for EOs and μg/ml for Tc.*

*C, Coridothymus capitatus; G, Eugenia caryophyllata; T, Thymus vulgaris; Tc, tetracycline; C-Tc, G-Tc, T-Tc, combination between the respective EO and tetracycline; MIC, minimum inhibitory concentration; FIC_A_, individual FIC of the EOs; FIC_B_, individual FIC of Tc; FICI, fractional inhibitory concentration index of the combination between each EOs and Tc.*

*Activity: A, antagonism; C, commutative effect; I, no interaction; S, synergy.*

### Combined Antimicrobial Effect of Tetracycline and Essential Oils

In the checkerboard assay, the combination of tetracycline with the three EOs, *C. capitatus*, *E. caryophyllata*, and *T. vulgaris* was tested (Table 3). The combinations resulted in the clear reduction of the MIC value for the antibiotic (from 256 to 4 μg/ml) for each *Salmonella* strain. Regarding the EOs, the MIC values, both in combination and alone, were the same in most of the strains. Only 19.3% (6/31) and 6.4% (2/31) of the strains exhibited different values of MICs for *C. capitatus*, which were, respectively, higher and lower than the EO alone. For *E. caryophyllata* EO in combination, 9.7% (3/31) and 9.7% (3/31) of the strains displayed the increase and the decrease, respectively, of the MIC value. Finally, for *T. vulgaris* EO in combination with the antibiotic, 6.4% (2/31) and 9.7% (3/31) of the strains had MIC values higher and lower, respectively, compared with the same EO alone.

Regarding the MBC assays, the behavior of the *Salmonella* strains in the presence of tetracycline was the same as that observed for the MICs. The MBC for each strain was 256 μg/ml when the antibiotic was applied alone, whereas the MBC reached 4 μg/ml in the presence of the combination of tetracycline and EOs (Table 3). Also, in this case, the combination of the antibiotic with the EOs displayed restoration of susceptibility to tetracycline in *Salmonella* isolates. MBCs of *C. capitatus*, *E. caryophyllata*, and *T. vulgaris* EOs showed a similar trend with respect to MICs, with the best bioactivity of *C. capitatus* compared with the other EOs.

In the checkerboard assay, the FIC index values were calculated by considering all the combinations of tetracycline with each EO in which there was no visible growth.

A synergistic effect (FICI ≤ 1.0) was detected in 6.5% (2/31), 9.6% (3/31), and 6.5% (2/31) of the strains, in the antibiotic with *C. capitatus*, *E. caryophyllata*, and *T. vulgaris* EOs, respectively. However, in most cases, the effect was found to be commutative (FICI = 1). In fact, the FICI values detected between tetracycline and *C. capitatus*, *E. caryophyllata*, and *T. vulgaris* EOs ranged between 1.02 and 2.0 for 90.3% (28/31), 80.6% (25/31), and 90.3% (28/31) of the strains, respectively. The indifferent effect (FICI > 1.0–2.0) was observed only in 16.1% (5/31) and 6.5% (2/31) of the strains in the presence of tetracycline with *C. capitatus* and *T. vulgaris* EOs, respectively. Finally, the antagonistic effect between the EOs and tetracycline was detected only in one *Salmonella* strain.

### Principal Component Analysis

**FIGURE 1:**
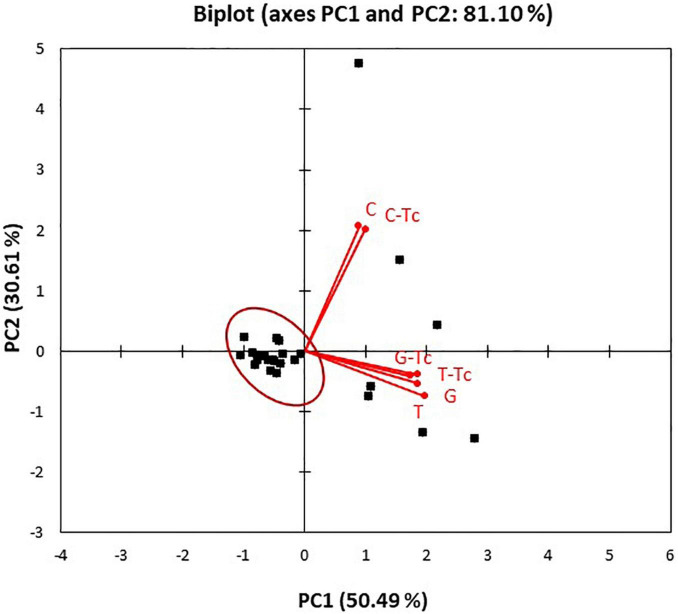
Principal component analysis (PCA) biplot (scores and loadings) based on antibacterial activity of *Coridothymus capitatus*, *Eugenia caryophyllata*, and *Thymus vulgaris* EOs alone or in combination with tetracyclines on the *Salmonella* strains. *C. capitatus* (C), *E. caryophyllata* (G), *T. vulgaris* (T), and tetracycline (Tc).

To assess the relationships between the type of treatment and bioactivity on the *Salmonella* strains, the dataset obtained from the MIC analysis was subjected to PCA. The PCA biplot (Figure 1) showed that the two principal components explained 81.10% of the total variance with the first axis (PC1) that contributed with 50.49% of the total variance and the second axis (PC2) with 30.61% of the total variance. The loading plot displayed the discrimination of the type of antimicrobial treatments along the PC2, showing the separation of *C. capitatus* from the other EOs, alone and in combination with tetracycline. The high value reached from each variable of the loading plot indicated the best bioactivity at the highest concentrations of the compounds. The score plot showed the distribution of the strains along the PC1, where they were mainly grouped in one cluster. As observed in the biplot, most of the *Salmonella* strains clustered with resistance to low concentrations of the various antimicrobial treatments (red circle). However, a smaller part of the strains (7/31) was distributed differently, showing a greater resistance to the EO treatment, closest to which it was positioned.

The PCA results confirmed the different effect of the EO treatments on the *Salmonella* strains, in particular, for *C. capitatus*, as already observed in MIC determinations (Table 3).

## Discussion

Antimicrobial compounds have been used to treat bacterial infections in humans and animals since the middle of the 20th century. The selective pressure has been causing the emergence of resistance, which is genetically encoded and subsequently inherited by the progeny of resistant pathogens (Munk et al., 2018). Due to the genetic nature of resistance and the ability to select resistant organisms through the use of antimicrobials in animals, their presence in animal products is considered a potential source of AMR in humans (Andrews and Ryan, 2015). Antibiotic-resistant bacteria have been found in food products (ready-to-eat, cooked meat), in cattle, poultry, swine, and goats in different stages of production (EFSA, 2021). In fact, in our study, all *Salmonella* spp. isolates showed phenotypic and/or genotypic resistance to at least one class of antibiotic examined. In detail, our results showed that *Salmonella* isolates were resistant to ampicillin, piperacillin, tetracycline, gentamicin, amikacin, and tobramycin (Table 2). The European Union Summary Report on AMR in zoonotic and indicator bacteria from humans, animals, and food in 2018/2019 showed resistance levels to ampicillin, sulfonamide, and tetracycline greater than 20%, and particularly, in Italy, 68–72% of the isolates were resistant to the abovementioned antibiotics. Ampicillin and tetracycline are commonly used in swine livestock as first-choice therapeutic antibiotics (Lekagul et al., 2019). They are also used as growth promoters, although in Europe, antibiotics have been banned as feed additives since 2006 (Lekagul et al., 2019). In particular, tetracyclines have been mainly used in animal health and in swine livestock, against Gram-positive and Gram-negative infections (Munk et al., 2018; Lekagul et al., 2019). Probably as a consequence, in our research, 31 *Salmonella* strains (15 *S.* Typhimurium, 3 Monophasic variant *S.* Typhimurium, 6 *S.* Enteritidis, 6 *S.* Rissen, and 1 *S.* Typhi) showed phenotypic resistance to tetracycline. According to our results, the AMR phenotype that is more present in the swine production chain is ampicillin, streptomycin, tetracycline, and chloramphenicol (Munk et al., 2018). Furthermore, all strains were sensitive to third-generation cephalosporins and fluoroquinolones, identified as “critically important antimicrobials” (CIA). In particular, all strains were sensitive to cefpodoxime, marbofloxacin, and enrofloxacin, while only 2.8% (1/36) was resistant to ceftiofur. In Europe, the resistance to third-generation cephalosporins is reported to be greater than 10%, and in Italy, more than 5% of the resistant strains were detected in animal samples (EFSA, 2021).

Regarding the antibiotic resistance genes, our results showed that most commonly detected were *parC* (100%), *catA* (94.4%), *nfsB* (86.1%), *nfsA* (77.7%), *blaTEM* (47.2%), *tetA* (47.2%), and *tetB* (41.6%). Our results are in agreement with other authors, who indicate that Italian pigs show the highest AMR levels in Europe (Munk et al., 2018). Other resistance genes, detected in a small proportion of samples (2.8%), were *tetC*, *aac(3)IV*, and *aadB*, as shown in Table 2.

The association of phenotypic resistance and the presence of AMR genes has been demonstrated to be variable among *Salmonella* serovars (Deekshit et al., 2012; McDermott et al., 2016). Similarly, divergent phenotypic and genotypic antimicrobial findings were obtained in our study. In fact, whereas phenotypic resistance to one or more antimicrobials was observed for both *S.* Derby (*n* = 2) and *S.* Rissen (*n* = 5), the corresponding resistance genes were not detected by PCR. On the contrary, for other strains, the opposite situation was observed. Deekshit et al. (2012), demonstrated that ubiquitous strains of non-typhoidal *Salmonella* can have silent AMR genes and that the correlation between phenotypic and genotypic resistance is not always possible (Deekshit et al., 2012). Some phenotypic and genotypic discrepancies observed may have been possible because not all the resistance genes were tested. Nevertheless, according to literature, some resistance mechanisms still remain unidentified (McDermott et al., 2016). For these reasons, when a resistance mechanism is detected in the genome, while the isolate is phenotypically susceptible, the interpretation criteria of the antibiogram may also be questioned (Lepuschitz et al., 2019).

Antibiotic resistance represents a current global concern, widespread in different fields of pharmaceutical sciences. As mentioned above, the possibility of combining antibiotics with EOs can be an alternative to overcome AMR in bacteria (Solarte et al., 2017). In veterinary clinical practice, data concerning EO treatments *in vitro* and *in vivo* do not draw a complete picture as in human medicine (Ebani and Mancianti, 2020). Nevertheless, the positive outcomes of EO treatments have been correlated with both their direct antimicrobial effects and their aspecific antioxidant and anti-inflammatory effects (Miguel et al., 2020), along with the immunomodulatory activity (Valdivieso-Ugarte et al., 2019).

Regarding our results (Table 3), all of the studied *Salmonella* isolates displayed MIC ≥ 256 μg/ml to tetracycline. Conversely, the three EOs and, in particular, *C. capitatus* and *T. vulgaris* EOs inhibited bacterial growth (Table 3). The *Salmonella* spp. strains were not able to grow even in the presence of the lowest concentrations of the two EOs (0.31 and 0.625 μl/ml), showing low levels of resistance to these compounds. Moreover, even in the presence of *E. caryophyllata* EO, the strains showed a reduction in growth capacity, as evidenced by the MIC values, although with values higher than the other two EOs (52% of strains showed MIC values of 20 μl/ml).

The antimicrobial activity of *C. capitatus*, *T. vulgaris*, and *E. caryophyllata* EOs can be attributed to the whole phytocomplex; nevertheless, the principal components carvacrol, thymol, and eugenol are known to exert antimicrobial effects. In detail, the mode of action of these natural compounds mainly involves the microbial membrane. The inhibitory activity of the natural compounds has been related to their hydrophobicity, which influences their partition in the cytoplasmic membrane (Lanciotti et al., 2004). The increased toxicity on the cytoplasmic membrane is directly correlated to the higher hydrophobicity level of the natural compound. Arfa et al. (2006) affirmed that carvacrol, due to its high hydrophobicity, exerts the highest antimicrobial activity. Contrarily, eugenol shows a lower efficacy that could be attributed to its lower hydrophobicity. Carvacrol is the major compound of *C. capitatus* EOs, the botanical species also called *Thymbra capitata* (Verdeguer et al., 2020). The best effectiveness of this EO, observed in terms of MIC values (Table 3) and confirmed by the different discrimination in PCA biplot (Figure 1), is probably due mainly to carvacrol. Carvacrol and thymol are characterized by the presence of a hydroxyl group and a system of delocalized electrons that are important for the antimicrobial activity of these phenolic compounds. This chemical structure allows carvacrol and thymol to act as proton exchangers, able to reduce the transmembrane gradient. The consequence is the collapse of the proton motive force and the depletion of the ATP pool, which can lead to cell death (Arfa et al., 2006).

The interactions between natural compounds, such as carvacrol and cell membranes, are described to affect both the lipid ordering and the bilayer stability, resulting in membrane integrity decrease (Arfa et al., 2006) and modification of the efflux pump activities (Bolla et al., 2011). Efflux is a mechanism that protects bacterial cells by expelling toxic compounds, such as antibiotics, before they can reach the intracellular targets (Davies, 1994). In Gram-negative bacteria, a tripartite efflux system is necessary to expel the drug to the outer medium: a protein localized in the cytoplasmic membrane, another in the periplasmic space, and a third in the outer membrane (de Sousa Oliveira et al., 2016). The efflux pumps are responsible for drug resistance in pathogenic bacteria, representing one of the main targets to overcome microbial resistance. Five families of membrane-spanning efflux proteins are recognized: major facilitators (MFs), small multidrug resistance (SMR), resistance nodulation cell division (RND), ATP-binding cassette (ABC), and multidrug and toxic compound extrusion (MATE) (Trifan et al., 2020). Active efflux systems have been commonly observed in the *Salmonella* genus and include *tetA* and *tetB*, the genes associated with tetracycline resistance (Frye and Jackson, 2013).

Miladi et al. (2017) observed that carvacrol, thymol, and eugenol act as efflux pump inhibitors in *S.* Typhimurium, causing the accumulation of the antibiotic, thereby acting synergistically. Synergy occurs when the combined effect of two or more substances is greater than the sum of the individual agents, in terms of enhanced therapeutic actions on the same target (Zhou et al., 2016). As demonstrated by our results (Table 3), synergistic effects between EOs and tetracycline were observed in a few cases and only when the FIC index was ≤1.0. Pirintsos et al. (2020) argued that the main reason for employing combinations of active substances, with synergistic interactions, is to reduce the administered amount of each compound and to increase the biological activity of a preparation/mixture against a specific target. Although the FICI classification did not highlight the synergy between the two types of antimicrobials, the effectiveness of the different compounds was evident. In fact, Table 3 displays the general decrease in tetracycline concentration (from 256 to 4 μg/ml) to which the *Salmonella* spp. strains were able to resist, only in combination with the EOs. This evidence suggests a restoration of susceptibility in *Salmonella* spp. to the antibiotic, as a consequence of the presence of EOs. As mentioned before, the inhibition of the efflux pumps by the EOs could cause antibiotic accumulation. In addition, the presence of EOs with a destabilizing effect on the bacterial membrane could facilitate antibiotic penetration into the cytoplasm and the easier reaching of target sites, such as the ribosome. In fact, tetracycline affects the 30S subunit of the bacterial ribosome, thus, inhibiting protein synthesis (de Sousa Oliveira et al., 2016).

The combinations of EOs and antimicrobial compounds could be an important instrument to reduce or reverse AMR (Trifan et al., 2020). The synergistic effect between EOs and antibiotics against multidrug-resistant bacteria has been described by other authors (Fadli et al., 2016). However, there is a lack of studies about the combination of EOs and tetracycline against multidrug-resistant *Salmonella* isolates (Trifan et al., 2020), in spite of the abundance of multiresistant strains and of the key role of tetracycline in animal husbandry.

## Conclusion

This study investigated a new approach to overcome multidrug resistance in *Salmonella* spp. isolated from the swine production chain. With this is mind, the combination of antibiotics and EOs was evaluated, by using *T. vulgaris*, *E. caryophyllata*, and *C. capitatus* EOs. Our results confirmed the evidence of the widespread AMR. In fact, *Salmonella* spp. exhibited a complex pattern of AMR, which underlines the need of new weapons to overcome multidrug resistance in the swine production chain. The combination of natural compounds with antibiotics represents a possible strategy. In fact, the most relevant result of the study was that the use of the EOs in combination with tetracycline showed the ability to restore the antibiotic effect of tetracycline in *Salmonella* strains. In spite of the importance of the topic, the studies related to the combinations of EOs and tetracyclines against *Salmonella* multidrug-resistant strains are lacking. In this still unexplored scenario, our results can represent a starting point. In this perspective, our research can be a piece of the puzzle in which the current dataset is not complete but is a starting point for further investigations. The potential future studies could evaluate the regulation of resistance genes following treatment with EOs and the investigation of the modulation of cell membrane proteins by proteomics approaches.

## Data Availability Statement

The data presented in the study are included in the article/supplementary material, further inquiries can be directed to the corresponding author.

## Author Contributions

AS, AP, and AV designed the study. CL, FM, and AF performed the experiments and wrote the manuscript. AS was responsible of the data validation. AP and AV gave important intellectual advice. All the authors checked, read, and approve the final version of manuscripts.

## Conflict of Interest

The authors declare that the research was conducted in the absence of any commercial or financial relationships that could be construed as a potential conflict of interest.

## Publisher’s Note

All claims expressed in this article are solely those of the authors and do not necessarily represent those of their affiliated organizations, or those of the publisher, the editors and the reviewers. Any product that may be evaluated in this article, or claim that may be made by its manufacturer, is not guaranteed or endorsed by the publisher.
